# The VEGA Tool to Check the Applicability Domain Gives Greater Confidence in the Prediction of In Silico Models

**DOI:** 10.3390/ijms24129894

**Published:** 2023-06-08

**Authors:** Alberto Danieli, Erika Colombo, Giuseppa Raitano, Anna Lombardo, Alessandra Roncaglioni, Alberto Manganaro, Alessio Sommovigo, Edoardo Carnesecchi, Jean-Lou C. M. Dorne, Emilio Benfenati

**Affiliations:** 1 Department of Biotechnology and Life Science, University of Insubria, Via Dunant 3, 21100 Varese, Italy; adanieli@uninsubria.it; 2 Laboratory of Environmental Toxicology and Chemistry, Department of Environmental Health Sciences, Istituto di Ricerche Farmacologiche Mario Negri IRCSS, 20156 Milano, Italy; erika.colombo@marionegri.it (E.C.); giuseppa.raitano@marionegri.it (G.R.); anna.lombardo@marionegri.it (A.L.); emilio.benfenati@marionegri.it (E.B.); 3 KODE Srl, Via Nino Pisano 14, 56122 Pisa, Italya.sommovigo@kode-solutions.net (A.S.); 4 European Food Safety Authority (EFSA), Via Carlo Magno 1A, 43126 Parma, Italy; edoardo.carnesecchi@efsa.europa.eu (E.C.); jean-lou.dorne@efsa.europa.eu (J.-L.C.M.D.)

**Keywords:** in silico models, new approach methodologies (NAMs), toxicological endpoints, applicability domain (AD), VEGA tool

## Abstract

A sound assessment of in silico models and their applicability domain can support the use of new approach methodologies (NAMs) in chemical risk assessment and requires increasing the users’ confidence in this approach. Several approaches have been proposed to evaluate the applicability domain of such models, but their prediction power still needs a thorough assessment. In this context, the VEGA tool capable of assessing the applicability domain of in silico models is examined for a range of toxicological endpoints. The VEGA tool evaluates chemical structures and other features related to the predicted endpoints and is efficient in measuring applicability domain, enabling the user to identify less accurate predictions. This is demonstrated with many models addressing different endpoints, towards toxicity of relevance to human health, ecotoxicological endpoints, environmental fate, physicochemical and toxicokinetic properties, for both regression models and classifiers.

## 1. Introduction

Confidence in using (quantitative) structure–activity relationship ((Q)SAR) models is a critical issue to increase their acceptability as new approach methodologies (NAMs) in next generation risk assessment (NGRA). The difficulty is establishing whether a given (Q)SAR model can be used for a specific substance of interest. A model is based on the information within its training set, and one may expect that a model built from specific substance classes may poorly predict properties for substances belonging to other classes. Thus, if a model has been developed for anilines, it is not reasonable to apply it to alcohols. The problem arises in the case of modern models, which are often built up on heterogeneous classes using a training set that may seem diverse but will never cover all possible chemical differences. Furthermore, there are often families of chemicals that are difficult to predict. One of the reasons for this is that some substances have a peculiar behavior that is poorly represented in the training set, so the model cannot obtain suitable information representing these substances’ particular effect, which is masked within the larger set of substances. Therefore, regulatory authorities require evaluating the applicability domain (AD) of the model, as in the European legislation on industrial substances (the Registration, Evaluation, Authorisation and Restriction of Chemicals—REACH—regulation) [1] and in the OECD principles for (Q)SAR [2].

From a chemometric point of view, the task of defining the AD depends on whether the prediction is an interpolation or an extrapolation [3,4]. Several approaches to evaluate the AD of (Q)SAR models have been presented [5,6,7,8,9]. Usually, the information on the training set is used to characterize the chemical diversity of the target substance and to verify whether the substance to be predicted is similar or not; chemical descriptors are used for this purpose [3]. Outliers related to the chemical space have been identified, also providing tools for building (Q)SAR models that cover the AD [6]. In some cases, the SMILES format is used to examine rare features of the molecule [10,11].

Some software programs for AD provide a binary outcome, so that the predictions are identified as either inside or outside the AD. This is the case of the Danish (Q)SAR Database [12]. The Toxicity Estimation Software Tool (T.E.S.T.) of the US Environmental Protection Agency (US-EPA) applies a similar binary outcome and filters predictions by considering whether the (Q)SAR predictions are inside or outside the AD. These two platforms and other commercial ones, such as Leadscope, feature a checklist of considerations for the AD [13,14]. In some cases, the AD is addressed specifically in relation to specific toxicity alerts [15].

Since different methods are used to measure the AD, the percentage of substances outside the AD varies, and in some cases, it can be as low as 2% [14,16].

The VEGAHUB [17] includes VEGA, which is a platform providing more than 100 (Q)SAR models, and other tools for prioritization, risk assessment, and read-across; users can download the software as an open-access resource. Over the last few years, the VEGAHUB has been used by the European Chemical Agency (ECHA) for screening substances that have been pre-registered under REACH [18]. VEGAGHUB is linked to the OECD QSAR Toolbox version 4.4 and is also available as a stand-alone tool for predictions within other platforms, such as AMBIT [19] and CCLIC [20]. 

For each (Q)SAR model, VEGA employs quantitative measurements to address the AD, composed of multiple factors. Basically, besides checking the chemical similarity between the target substance and the substances in the training set, VEGA makes additional checks, specific to the endpoint and the algorithm. In practice, several checks are performed and the algorithm provides quantitative results. Predictions on the most similar substances are used to assess whether the prediction is reliable for the target substance. The ad hoc software checks whether the predictions of substances similar to the target one are correct. The experimental values for the most similar substances are then compared with the predicted value of the target substance. In this case, the software compares the agreement between the two values and any potential inconsistencies are indicated to the user. This is intended to help the user specifically address certain points; since the process is automated, it allows to filter out predictions with doubts related to the AD. Specific features regarding AD measurements within VEGA have been discussed elsewhere in several studies [21,22,23,24,25,26,27,28,29].

Overall, the AD tool within VEGA has been shown to provide satisfactory results. In this work, the assessment of the AD tool within VEGA was applied to a range of models of relevance to human health, ecotoxicity, environmental fate, physicochemical and toxicokinetic properties for both regression models and classifiers [30].

## 2. Results

The use of the (Q)SAR models is still limited and one of the reasons behind such limited use lies in the fact that users are not always confident in the prediction reliability of such in silico models. Notwithstanding, several means are available to assess the reliability of (Q)SAR model predictions. The first approach includes statistics related to the model outputs based on the whole set of available compounds (including training and test sets) used to build the model. This assessment provides an optimized evaluation of the model; it is probable that when the model is applied to new substances, predictions may not perform as well as the results based on the whole set, particularly because the results based on the substances in the training set are “facilitated” since they are inside the model. Furthermore, such a generic evaluation is based on a population of substances; however, predictions for the target compound may be different from the average results for the whole population.

A second approach is to assess the results of the test set and to constitute a sounder procedure, since the statistical outputs reflect predictions for substances that are external to the training set; this case is closer to the practical use of the model. Appropriate measurements using internal validation procedures are also a useful complimentary procedure. In this case, more confidence in the results can be obtained; still, uncertainty in the prediction performance for the target substance may arise. The third approach relies on the use of a tool that allows the assessment of the AD of the model, filtering results that are outside the AD; this method is available in some in silico platforms.

One key issue is to assess how effective these tools are for AD evaluation so that the most reliable predictions can be identified. In this case, the evaluation requires classifying a substance as either inside or outside the AD, while the algorithm at the basis of these tools refers to a distance, which is not a categorical entity; thus, arbitrary thresholds are applied.

The fourth method relies on applying a quantitative measurement of the AD (such as the Applicability Domain Index, ADI) and refers to the one described and discussed here for the VEGA tool (see Section 4 “Materials and Methods”). The aim is to assess whether the tool implemented in VEGA for the measurement of the AD can identify predictions which may be inconsistent. Here, the ADI is calculated using a set of substances never used to build up the model. Finally, a fifth approach is also available to assess prediction accuracy and relies on full evaluation of all elements provided by VEGA, such as similar substances and information on the mechanism(s) associated with predicted endpoints; currently, this process requires manual implementation.

The use of the ADI tool within VEGA has a range of advantages as follows:

1. Allowing the identification of issues related to prediction accuracy and providing the user with an opportunity for thorough analysis.
2. Allowing identification of mechanisms associated with structural features of the substances.
3. Analyzing similar substances through a read-across approach.
4. Filtering substances with more reliable predictions, to be used in batch mode for a range of substances.

The first three advantages refer to the use of in silico models within a weight-of-evidence (WoE) approach following the scheme provided in the EFSA Guidance on WoE [31] and further detailed for non-testing methods elsewhere [32]. The user should evaluate all three lines of evidence specified in the first three points discussed above: the prediction, the reasoning, and the experimental evidence. What VEGA provides should be evaluated in an integrated way. If the ADI value is low due to the presence of similar substances with conflicting results that affect the ADI but are irrelevant because they contain fragments of an adverse effect absent in the target substance, the user may disregard these substances and consider the prediction reliable, even if the ADI tool serves warnings. Conversely, if there is a very similar substance with a property value conflicting with the predictions, this may over-rule such prediction and the ADI will automatically indicate the issue.

In this study, the use of the ADI tool is described to identify more reliable predictions, which is useful in addressing many substances. Supplementary Materials reports the details of the calculations of all the statistical parameters. Below, we reported only the most representative parameters for classification and regression models, to compare the overall performance in a simplified way.

**Figure 1:**
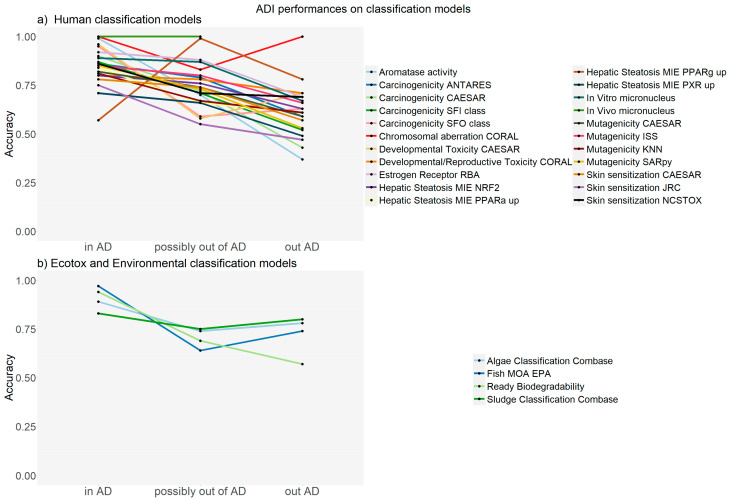
Accuracy of the classification models for the substances in the test set for human toxicity, ecotoxicity and environmental properties.

Figure 1 shows the statistical results, expressed as accuracy, for the substances in the test set, according to the classification models towards human toxicity, ecotoxicity, and environmental properties. In practice, the ADI can recognize potentially inconsistent results, and predictions in AD have the highest values. Figure 1a illustrates the prediction accuracy for human toxicological endpoints related to relevant in silico models. The predictions in the AD have the highest value; the only exception is the model for the molecular initiating event for PPAR alpha. For the CORAL model predicting chromosomal aberration, satisfactory results are shown for predictions that are also outside the AD. For two models, the CAESAR model for developmental toxicity and carcinogenicity oral classification, the values outside the AD are somehow better than the values of the predictions potentially outside the AD; but regardless, the predictions in the AD are always the better ones. Overall, the predictions potentially outside the AD are still satisfactory, while the predictions outside the AD are often less reliable.

**Figure 2:**
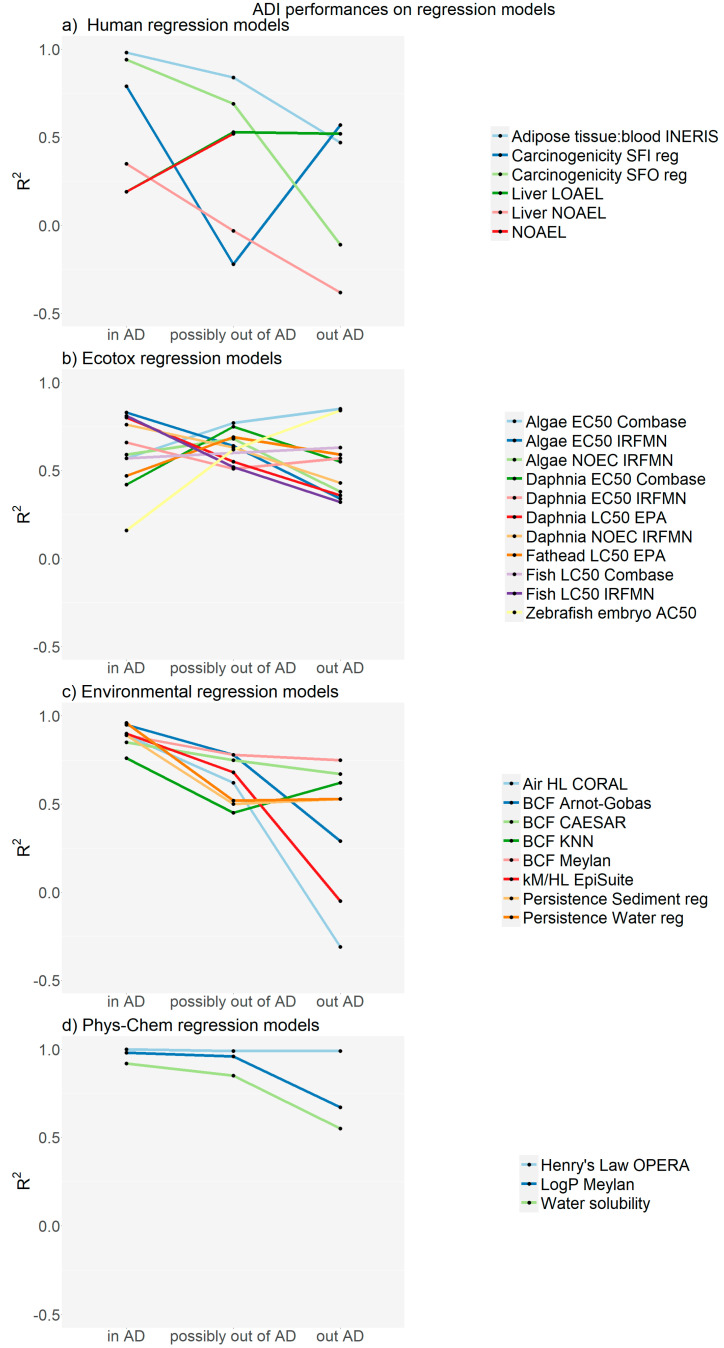
Correlation coefficients (R2) for (Q)SAR models for the substances in the test set for human toxicity, ecotoxicity, environmental/toxicokinetic and physicochemical properties.

Figure 2 shows the R2 related to endpoint predictions of the test set for the quantitative models towards human toxicity, ecotoxicity, environmental/toxicokinetic and physicochemical properties. In this case too, the use of ADI can identify potential issues with prediction correctness. As expected, quantitative models are generally more complex compared to classifiers, and so the results are not always ideal, mainly for the most complex endpoints, such as human toxicology and ecotoxicology. For many of these models for which no robust support from an ADI perspective was concluded, the input data included tests on few substances in the test set, and most often fewer than ten. This is the case for the three (Q)SAR models predicting LOAEL/NOAEL relevant to human toxicity (Figure 2a). When considering models predicting ecotoxicological properties (Figure 2b), predictions from the zebrafish embryo toxicity model did not perform very well and this can be rationalized by the fact that only seven molecules have been used. Hence, from a statistical point of view, more substances would need to be tested. For the other models too, only a few substances were used, and more substances would be required to get meaningful statistics. Thus, results are poor for the COMBASE models, particularly towards Daphnia and the EPISuite model for fish acute toxicity (as implemented in VEGA). Figure 2c illustrates the results for the environmental and toxicokinetic properties in fish. In this case, the statistics are satisfactory if the results are within the AD, with R2 values from 0.76 to 0.96. If the predictions are potentially out of the AD, the prediction correctness is weaker, and worse if the predictions are outside the AD. It is easier to model these properties because they are associated with less complex processes compared to those discussed above.

Figure 2d shows the prediction results for physicochemical properties and, for these substances within the ADI, predictions are excellent since these properties are relatively simple to model. These predictions are also satisfactory for substances potentially outside the AD, but the performance is weaker when the predictions are outside the AD.

### 2.1. Examples

To show the use of the ADI, two examples are reported below, one with a high ADI value and one with a low value. The outputs of the models are reported in the Supplementary Materials (Trifluralin_NOAEL_LIVER_CORAL.pdf and Diethyl(nitroso)amine_HENRY_OPERA.pdf).

#### 2.1.1. Trifluralin

Trifluralin, an herbicide, was predicted with the Liver NOAEL (CORAL) 1.0.1 model using the SMILES O=[N+]([O-])c1cc(cc(c1N(CCC)CCC)[N+](=O)[O-])C(F)(F)F. The model predicts a NOAEL of 2.34 log units (around 221 mg/kg bw) with an ADI of 1.

Table 1 reports the parameters that compose the ADI. The similarity index is high and, indeed, the two most similar chemicals found in the training set have a similarity higher than 0.96. The third similar substance has a high similarity (0.895) too, but it was not considered in the ADI calculation (which is based on the first two similar chemicals only). Observing the predicted and experimental values of these two similar substances, the second has the highest difference, but in general, they are around 0.5 log units. The last two parameters indicate that Trifluralin has no rare or unknown groups and that the descriptors’ values are in the range of the descriptors of the entire training set.

The good reliability of this prediction is confirmed by the experimental value available for Trifluralin: 2.19 log units (around 154 mg/kg bw).

#### 2.1.2. Diethyl(nitroso)amine

Diethyl(nitroso)amine is an industrial chemical with a predicted Henry’s law constant of −4.94 log atm-m3/mole (see Table 1 for details on the ADI).

In this case, the ADI is based on the first three similar chemicals, that have a sufficiently high similarity (similarity between 0.76 and 0.803). They are correctly predicted (accuracy index of 0.452), even if the first similar chemical has a moderate error (0.765). The prediction for the Diethyl(nitroso)amine is not concordant with the experimental values of the similar substances (especially similar 1 and 3). This may be due to the structural differences. Indeed, none of the similar substances have the nitrosoamine group. The presence of unknown fragments is also highlighted by the ACF index.

The experimental value confirms the low quality of this prediction (−5.44 log atm-m3/mole).

## 3. Discussion

There are several AD tools available in the various software platforms. In this work, a systematic process has been described and investigated their effectiveness with regards to inconsistencies in predictions. The use of the ADI tool within VEGA allows supporting expert judgment without replacing it and the three categories of ADI values (high, moderate, and low) have different statistical qualities. Indeed, this is particularly helpful in the case of prediction inaccuracies with a high ADI or when accurate predictions are reported with a low ADI. However, the prevalence of these predictions inconsistencies is higher for substances with a low ADI. Confidence in these results is related to linking predictions to available information for the substances that are at the basis of the model, i.e., those in the training set. The composite ADI tool proved efficient in capturing this information. The AD should not be evaluated simply on the basis of the chemical information, and the ADI tool can detect prediction issues resulting from the in silico model itself that is specific to a certain endpoint.

The advantage of the ADI tool implemented in VEGA lies in the fact that it is convenient to use VEGA for models available also within other platforms. For instance, VEGA contains the same (Q)SAR model for mutagenicity (Ames test) implemented in Toxtree. However, Toxtree does not provide an evaluation of the AD and the user cannot identify the most similar substance, which is useful for evaluation and read-across procedures. Other models include those available in EPISuite for BCF for which the AD must be analyzed manually, which is quite a complex process.

This manuscript highlighted that it is possible to identify prediction accuracy for a range of models resulting in a range of statistical quality, depending on the ADI value. What is typically described for the results of a model are the statistical results, for instance on the training and test sets which are provided at the level of the whole population of chemicals. In our case, the statistical quality of the results for substances with a high ADI was higher in most cases. Thus, if the ADI is high, the expectation is that the prediction accuracy of the model will be higher than those observed on the whole population of substances. In a few cases, the ADI does not improve prediction accuracy. In this case, sound statistical values for prediction accuracy are reflected at the population level, or one may expect even lower prediction accuracy for low ADIs. In cases where the prediction of the (Q)SAR model is not satisfactory, applying read-across is recommended. This can be performed by considering similar substances provided by VEGA using ToxRead as another tool present in VEGAHUB and offering tens of modules for different endpoints, or using the VERA tool [33].

## 4. Materials and Methods

### 4.1. Applicability Domain Index within VEGA

Since in silico models, including those available within the VEGAHUB, are constructed on three pillars, namely endpoint, chemical information, and the algorithm providing predictions, the applicability domain index (ADI) requires a thorough assessment on these three components. Depending on the specific model, there are some specific components of the ADI, for instance, if the model is a regression model or a classifier.

The chemical information is assessed by considering the chemical similarity. This is measured according to several parameters and provides values ranging from 0 to 1 (1 indicates identity) [34]. Such values can be used to assess how similar the substances in the training set are. The chemical similarity, as in the case of all similarities, is not an objective measurement, and there are many possible ways to measure it. For this reason, VEGA provides images of the six most similar substances, so that users can weigh the evidence depending on the context of the chemical assessment. Another parameter related to the chemical information within the ADI is the chemometric check, which allows users to assess whether the target substance has descriptors outside the range of the descriptor values of the substances in the training set. In all cases, the range of the molecular weights is checked, even if the molecular weight is not one of the available descriptors. In addition, the software assesses whether there are rare fragments in the target substance; for this purpose, VEGA uses atom-centered fragments.

There are three components of the ADI related to specific endpoints, namely prediction accuracy, concordance between the predicted value for the target substance and the experimental values, and presence of fragments associated with outliers for a given fragment:

1. VEGA checks the accuracy of the predictions for similar substances. In this case, the predicted value of the similar substance is compared with the experimental value. If the value is a label, such as mutagenic or not, the comparison is provided instantly. In the case of quantitative values, the software considers the quantitative differences across substances and an additional factor reports whether the difference in the prediction is very large or not.
2. Concordance between the predicted value for the target substance and the experimental value of the similar compound is another very important parameter for the ADI. In this case, the prediction (i.e., the prediction accuracy of the in silico model) can be related to the “read-across” use of the VEGA output, showing the most similar substances. In particular, if predictions are different from the experimental values of similar substances, this poses a question, while if there is agreement, this increases the ADI. If the model provides structural alerts, VEGA provides an additional check and indicates whether for a similar substance, one or more structural alerts are present, and if such a structural alert is present in the target substance too. This is a valuable piece of information, highlighting to the user, for instance, that there is a structural alert only for a similar substance. Thus, the user can decide to disregard such a similar substance as non-relevant.
3. The last component of the ADI regarding the specific endpoint is the presence of fragments associated with outliers for that endpoint. This component is present only for a few models, where the model poorly predicted a particular chemical family.

In addition, two components are associated with the algorithm, namely uncertainty of the model for specific endpoints and the sensitivity of the prediction for a given descriptor reported for the target compound. VEGA modifies the descriptor value for a small value and checks whether this causes a large difference in the predicted value.

Based on all these components, the overall ADI value is calculated, and VEGA reports the values for each component of the ADI and the overall sum. To help the user, a graphical symbol is shown and indicates warnings qualified as “no”, “moderate”, or “strong”.

The components are measured for the two or three most similar compounds, even though the software shows the six most similar compounds.

### 4.2. Categories of ADI Values

An ADI serves as a continuous quantitative value and, to help the user, VEGA gives an indication regarding the quality of this value, indicating whether the prediction seems reliable, moderate or of low quality. The main purpose of the ADI should be to highlight the sources of concern and the severity of those concerns rather than simply to identify good predictions. A low ADI highlights that the user should carefully check the issues indicated by the ADI. In contrast, a moderate ADI highlights that some issues require further assessment. Overall, all predictions should be assessed thoroughly, including those with satisfactory results, and the ADI provides a useful tool to the user to do this.

When many predictions are available, the ADI supports a classification of the results according to their probable reliability. Thus, already in the summary of the output of the prediction, VEGA provides the prediction and this evaluation, presented with one, two, or three stars. In our experience, the ADI is high if it is >0.85, moderate if it is between 0.75 and 0.85, and low if it is <0.75. These are general values and may vary according to the endpoint. All the threshold values for the ADI and its individual components, which are model specific, are described in the model user guide.

### 4.3. Modified ADI

Results from VEGA model predictions are used to exploit all three lines of evidence: (1) prediction; (2) similar substances to be used for read-across; and (3) reasoning with regards to the potential mechanism of toxicity for a given endpoint, for instance, structural alerts.

These lines of evidence have already been introduced above in an implicit manner while discussing the accuracy of the prediction in the ADI, the concordance (evidence of the experimental data used in the read-across strategy), and the reasoning, as indicated by the presence of structural alerts. Since VEGA is a tool for the evaluation of chemicals, the evidence of experimental data (all data, in the training and the test sets) is very important. Thus, particularly for the purpose of read-across, VEGA shows the most similar substances both in the training and test sets.

Other software systems use a different perspective. For instance, T.E.S.T., a valuable platform, highlights the statistical quality of the results when considering the substances in the training or the test set separately. The quality of the predictions for the substances in the test set is valuable for evaluating the statistical quality of the model. The assessment within VEGA is not focused on the (Q)SAR model itself, but uses all lines of evidence, including read-across and reasoning [32]. However, for the purposes of this study, VEGA has been modified, so that the ADI is calculated only for the training set. This made it possible to calculate the results for new substances, avoiding the risk that the software finds the target substance in the test set. Hence, only the substances in the training set are used in our study for the AD assessment so that the results can be examined for substances that were not used to build up the model.

### 4.4. Test Set

To assess the results of the (Q)SAR models in VEGA, only substances in the test set were examined, as described in Section 4.3. However, some models in VEGA are not of statistic nature, since they are not based on a training set, but on expert-based rules. For instance, this is the case for models derived from Toxtree based on mutagenicity rules, or on Cramer classes. For these reasons, we do not have the assessment based on the ADI for all models.

In other cases, the number of substances in the test set was quite small, so we looked for substances in other sources. For statistical analysis, only the molecules outside the training set of each model were considered. This was the case for the assessment of the BCF model performance (Arnot–Gobas) and the BCF model (KNN/Read-Across), for which 1129 compounds were collected from the literature as an external dataset [35].

For the skin sensitization models (CAESAR and IRFMN/JRC), a dataset of 623 compounds was used with univocal local lymph node assay (LLNA) assessment. The data were collected (removing duplicates) from several public sources [36,37,38,39,40,41,42].

For the mutagenicity (Ames) endpoint, the data were selected from a large dataset (about 18,000 compounds) containing public and proprietary data [30,43].

### 4.5. Performance Parameters

The performance of the models was evaluated on the basis of accuracy or R2 for classifier or regression models, respectively. More detailed information about these and other parameters can be found in the Supplementary Materials (Supplementary_Material.xlsx).

## 5. Conclusions

This manuscript investigated the systematic use of the ADI from the VEGA tool to gain confidence in using (Q)SAR models as part of NAM batteries within NGRA of chemicals. This tool offers a powerful way to identify critical issues for the specific substance and model. VEGA provides not only the predicted value, but also many more parameters that should be thoroughly assessed. The information includes the prediction accuracy itself, the presence of similar substances, and the elements for reasoning in relation to mechanisms of toxicity. All these elements are provided and should be evaluated. The ADI tool serves as a relevant tool to assess these multiple elements and provided reliable results, increasing confidence in using such models. Overall, the ADI is a quantitative value, but for convenience, it can also be represented graphically as categories using stars as a graphical metric of prediction reliability. The warning messages identified through the ADI analysis help to identify the critical aspects that the user should carefully assess. Finally, when the user is working in batch mode, the results with a higher ADI are preferable, and this approach can be used to filter results. Since the tool is transparent, sophisticated, and detailed, it provides a sound way to obtain accessible, intelligible, useful, and assessable results.

## Figures and Tables

**Table 1:** The details on the ADI for the two examples.

Parameters	Trifluralin	Diethyl(nitroso)amine
ADI	1.000	0.309
Similarity index	0.975	0.773
Accuracy index	0.486	0.452
Concordance index	0.470	1.860
Max error index	0.507	0.765
Descriptors range check	True	-
ACF index	1.000	0.400

## Data Availability

The data presented in this study are openly available within the VEGA platform (https://www.vegahub.eu/portfolio-item/vega-qsar/ accessed on 6 February 2023).

## Supplementary Materials

The following supporting information can be downloaded at: https://www.mdpi.com/article/10.3390/ijms24129894/s1, Supplementary_Material.xlsx (Table S1—Statistics of regression models, Table S2—Statistics of binary classification models, Table S3—Statistics on multiclass models); Trifluralin_NOAEL_LIVER_CORAL.pdf; Diethyl(nitroso)amine_HENRY_OPERA.pdf.
