# Supplementary material for: The VEGA Tool to Check the Applicability Domain Gives Greater Confidence in the Prediction of In Silico Models
Source: Int J Mol Sci. 2023 Jun 8;24(12):9894. doi: 10.3390/ijms24129894 (PMC10298077; doi:10.3390/ijms24129894)
Supplement: Supplementary file 1 [file ijms-24-09894-s001.zip › Diethyl(nitroso)amine_HENRY_OPERA.pdf]

VEGA QSAR MODELS

Report

Prediction and Applicability Domain analysis for models:

Henry's Law model (OPERA) 1.0.1

Core version: 1.3.14

1. Prediction Summary

Prediction for compound Molecule 0 -

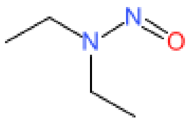

Prediction:

Reliability:

Prediction is -4.9375 log atm-m3/mole, but the result may be not reliable. A check of the information given in the following section should be done, paying particular attention to the following issues:

- similar molecules found in the training set have experimental values that disagree with the predicted value
- the maximum error in prediction of similar molecules found in the training set has a moderate value, considering the experimental variability
- a prominent number of atom centered fragments of the compound have not been found in the compounds of the training set or are rare fragments (2 unknown fragments found)

Compound: Molecule 0
Compound SMILES: O=NN(CC)CC
Experimental value: -
Predicted Henry's law [log atm-m3/mole]: -4.9375
Reliability: The predicted compound is outside the Applicability Domain of the model
Remarks:
none

3.1 Applicability Domain:
Similar Compounds, with Predicted and Experimental Values

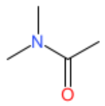

Compound #1

CAS: 127-19-5
Dataset id:239 (Training Set)
SMILES: O=C(N(C)C)C
Similarity: 0.803
Experimental value : -7.883
Predicted value : -7.118

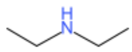

Compound #2

CAS: 109-89-7
Dataset id:188 (Training Set)
SMILES: N(CC)CC
Similarity: 0.761
Experimental value : -4.593
Predicted value : -5.048

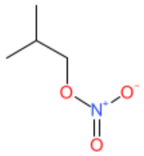

Compound #3

CAS: 543-29-3
Dataset id:287 (Training Set)
SMILES: O=[N+]([O-])OCC(C)C
Similarity: 0.76
Experimental value : -2.648
Predicted value : -2.786

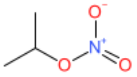

Compound #4

CAS: 1712-64-7
Dataset id:334 (Training Set)
SMILES: O=[N+]([O-])OC(C)C
Similarity: 0.755
Experimental value : -2.793
Predicted value : -2.785

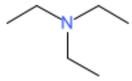

Compound #5

CAS: 121-44-8
Dataset id:220 (Training Set)
SMILES: N(CC)(CC)CC
Similarity: 0.754
Experimental value : -3.827
Predicted value : -3.979

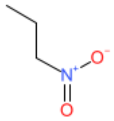

Compound #6

CAS: 108-03-2
Dataset id:157 (Training Set)
SMILES: O=[N+]([O-])CCC
Similarity: 0.75
Experimental value : -4.06
Predicted value : -4.576

3.2 Applicability Domain:

Measured Applicability Domain Scores

✗	Global AD Index<br>AD index = 0.309<br>Explanation: The predicted compound is outside the Applicability Domain of the model.
✓	Similar molecules with known experimental value<br>Similarity index = 0.773<br>Explanation: Strongly similar compounds with known experimental value in the training set have been ..
✓	Accuracy of prediction for similar molecules<br>Accuracy index = 0.452<br>Explanation: Accuracy of prediction for similar molecules found in the training set is good..
✗	Concordance for similar molecules<br>Concordance index = 1.86<br>Explanation: similar molecules found in the training set have experimental values that disagree with the predicted value..
⚠	Maximum error of prediction among similar molecules<br>Max error index = 0.765<br>Explanation: the maximum error in prediction of similar molecules found in the training set has a moderate value, considering the experimental variability..
✗	Atom Centered Fragments similarity check<br>ACF index = 0.4<br>Explanation: a prominent number of atom centered fragments of the compound have not been found in the compounds of the training set or are rare fragments (2 unknown fragments found)..

Symbols explanation:

✓ The feature has a good assessment, model is reliable regarding this aspect.

⚠ The feature has a non optimal assessment, this aspect should be reviewed by an expert.

✗ The feature has a bad assessment, model is not reliable regarding this aspect.

4.1 Reasoning:
Relevant Chemical Fragments and Moieties

(Molecule 0) Reasoning on rare and missing Atom Centered Fragments .
The following Atom Centered Fragments have been found in the molecule, but they are not found or rarely found in the model's training set:

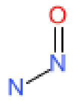

Fragment defined by the SMILES: NN=O
The fragment has never been found in the model's training set

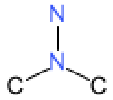

Fragment defined by the SMILES: CN(C)N
The fragment has never been found in the model's training set
