# Supplementary material for: The VEGA Tool to Check the Applicability Domain Gives Greater Confidence in the Prediction of In Silico Models
Source: Int J Mol Sci. 2023 Jun 8;24(12):9894. doi: 10.3390/ijms24129894 (PMC10298077; doi:10.3390/ijms24129894)
Supplement: Supplementary file 1 [file ijms-24-09894-s001.zip › Trifluralin_NOAEL_LIVER_CORAL.pdf]

VEGA QSAR MODELS

Report

Prediction and Applicability Domain analysis for models:

Liver NOAEL (CORAL) 1.0.1

Core version: 1.3.14

1. Prediction Summary

Prediction for compound Molecule 0 -

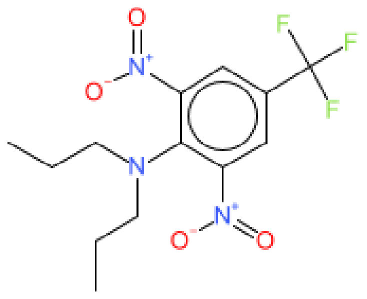

Prediction: Reliability:

Prediction is 2.3449, the result appears reliable. Anyhow, you should check it through the evaluation of the information given in the following sections.

Compound: Molecule 0
Compound SMILES: O=[N+]([O-])c1cc(cc(c1N(CCC)CCC)[N+](=O)[O-])C(F)(F)F
Experimental value: -
Liver NOAEL [log(mg/kg bw)]: 2.3449
Reliability: The predicted compound is into the Applicability Domain of the model
Remarks:
none

3.1 Applicability Domain:

Similar Compounds, with Predicted and Experimental Values

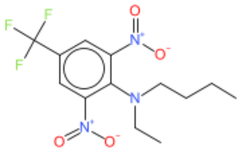

Compound #1

CAS: N.A.
Dataset id:173 (Training Set)
SMILES: O=[N+]([O-])c1cc(cc(c1N(CC)CCCC)[N+](=O)[O-])C(F)(F)F
Similarity: 0.99
Experimental value : 2.718
Predicted value : 2.253

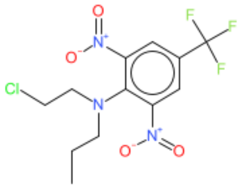

Compound #2

CAS: N.A.
Dataset id:56 (Training Set)
SMILES: O=[N+]([O-])c1cc(cc(c1N(CCC)CCCl)[N+](=O)[O-])C(F)(F)F
Similarity: 0.961
Experimental value : 1.778
Predicted value : 2.285

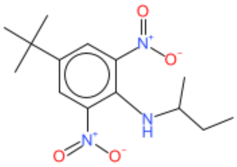

Compound #3

CAS: N.A.
Dataset id:129 (Training Set)
SMILES: O=[N+]([O-])c1cc(cc(c1(NC(C)CC))[N+](=O)[O-])C(C)(C)C
Similarity: 0.895
Experimental value : 1
Predicted value : 2.256

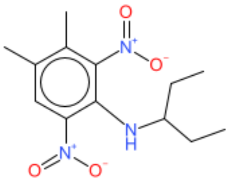

Compound #4

CAS: N.A.
Dataset id:107 (Training Set)
SMILES: O=[N+]([O-])c1cc(c(c(c1(NC(CC)CC))[N+](=O)[O-])C)C
Similarity: 0.891
Experimental value : 1.593
Predicted value : 2.265

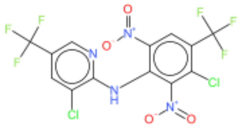

Compound #5

CAS: N.A.
Dataset id:160 (Training Set)
SMILES: O=[N+]([O-])c2cc(c(c(c2(Nc1ncc(cc1Cl)C(F)(F)F))[N+](=O)[O-])Cl)C(F)(F)F
Similarity: 0.766
Experimental value : 0.58
Predicted value : 1.994

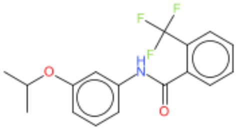

Compound #6

CAS: N.A.
Dataset id:139 (Training Set)
SMILES: O=C(Nc1cccc(OC(C)C)c1)c2ccccc2C(F)(F)F
Similarity: 0.764
Experimental value : 1.643
Predicted value : 2.077

3.2 Applicability Domain:
Measured Applicability Domain Scores

Global AD Index<br>AD index = 1<br>Explanation: The predicted compound is into the Applicability Domain of the model.
Similar molecules with known experimental value<br>Similarity index = 0.975<br>Explanation: Strongly similar compounds with known experimental value in the training set have been ..
Accuracy of prediction for similar molecules<br>Accuracy index = 0.486<br>Explanation: Accuracy of prediction for similar molecules found in the training set is good..
Concordance for similar molecules<br>Concordance index = 0.47<br>Explanation: Similar molecules found in the training set have experimental values that agree with the predicted value..
Maximum error of prediction among similar molecules<br>Max error index = 0.507<br>Explanation: the maximum error in prediction of similar molecules found in the training set has a low value, considering the experimental variability..
Model's descriptors range check<br>Descriptors range check = True<br>Explanation: descriptors for this compound have values inside the descriptor range of the compounds of the training set..
Atom Centered Fragments similarity check<br>ACF index = 1<br>Explanation: all atom centered fragment of the compound have been found in the compounds of the training set..

Symbols explanation:

The feature has a good assessment, model is reliable regarding this aspect.

The feature has a non optimal assessment, this aspect should be reviewed by an expert.

The feature has a bad assessment, model is not reliable regarding this aspect.
